# Multiple cross displacement amplification-based lateral flow biosensor for rapid and sensitive detection of *Helicobacter pylori*

**DOI:** 10.3389/fcimb.2024.1396330

**Published:** 2024-11-14

**Authors:** Yanfei Chen, Juan Zhou, Jiao Wang, Xi He, Xiaolan Huang, Fei Xiao, Nan Jia, Yi Wang, Xuemei Zhong

**Affiliations:** ^1^ Department of Gastroenterology, Affiliated Children’s Hospital, Capital Institute of Pediatrics, Beijing, China; ^2^ Experimental Research Center, Capital Institute of Pediatrics, Beijing, China

**Keywords:** *Helicobacter pylori*, multiple cross displacement amplification, lateral flow biosensor, HP-MCDA-LFB, infection

## Abstract

*Helicobacter pylori* (*H. pylori*, HP), recognized globally as one of the most widespread bacteria, serves as primary etiological agent for numerous gastroduodenal diseases, highlighting the urgent need to develop rapid and sensitive diagnostic method for *H. pylori* infection. Here, we devised a new diagnostic test that merged multiple cross displacement amplification (MCDA) with nanoparticle‐based lateral flow biosensor (LFB), termed HP‐MCDA‐LFB, to facilitate the rapid and sensitive detection of *H. pylori*. The whole detection workflow, which includes stages such as DNA template extraction (~15 min), MCDA pre-amplification (~40 min), and result readout (~2 min), was efficiently completed within 1 h. After optimization, the HP-MCDA-LFB assay demonstrated remarkable sensitivity in detecting *H. pylori*, with a detection threshold as low as 60 fg of genomic DNA (~56 copies) per microliter. Furthermore, the HP-MCDA-LFB assay also achieved a perfect specificity rate of 100%, exhibiting no cross-reactivity with non-*Helicobacter* isolates. Particularly, the clinical feasibility of HP-MCDA-LFB assay was validated using 40 antral mucosa samples, among which 17 tested positive for *H. pylori*, which was in complete agreement with the results obtained from the rapid urease test. In conclusion, the HP‐MCDA‐LFB method developed in this study is a rapid, sensitive, and specific method for diagnosing *H. pylori* infection, indicating great potential for *H. pylori* eradication therapy.

## 1 Introduction

*Helicobacter pylori* (*H. pylori*, HP), a prevalent cause of chronic bacterial infections in humans, is responsible for various types of gastrointestinal disorders, including chronic gastritis, peptic ulcer disease (PUD), gastric cancer, and gastric-mucosa-associated lymphoid tissue lymphoma (Malfertheiner et al., 2017). Beyond these ailments, *H. pylori* has also been associated with non-digestive issues in children, such as short stature, refractory iron deficiency anemia, malnutrition, and halitosis (Jones et al., 2017). The eradication of *H. pylori* plays a key role in the management of these clinical infestations, demonstrating the urgent need for accurate detection methods for *H. pylori*, especially the methods with sensitivity and specificity exceeding 90% (Xiang et al., 2022).

The current diagnostic landscape for *H. pylori* infection in clinical settings is divided between non-invasive methods like the ^13^C-urea breath test (UBT), stool antigen test (SAT), and serological test, and invasive approaches that include bacterial culture, histopathological examinations, and the rapid urease test (RUT). These methods have trade-offs in terms of simplicity, speed, and accuracy (Malfertheiner et al., 2017; Xiang et al., 2022). Non-invasive tests are generally safer for patients but often compromise on detection sensitivity and specificity. Because of its high accuracy, the UBT is currently recommended as the best method for test of *H. pylori* infection. However, this test also has its drawbacks, such as its high cost and need for mass-spectrometric analysis, which limited the application in remote or resource-limited settings. Moreover, the high risk of false-positive and false-negative discounted the wide employment of UBT. The SAT is also a preferred method for epidemiological study and screening tests because it is noninvasive, cost effective, and easy to perform. However, if patients are subjected to proton pump inhibitor (PPI) treatment, the sensitivity of the tests decreased (Malfertheiner et al., 2017). Invasive methods can offer higher accuracy but require gastric biopsy samples, which necessitate endoscopic intervention (Atkinson and Braden, 2016; Chey et al., 2017; Chey and Wong, 2007; Hunt et al., 2011). Despite their reliability, bacterial culture and histopathological exams are hampered by their long turnaround times, complexity, and demand for skilled personnel, which constrain their widespread use and effectiveness in *H. pylori* detection, delaying eradication efforts (Xiang et al., 2022). Thus, easy-to-operation, simple, rapid, and sensitive methods for *H. pylori* diagnosis are yet to be developed.

Nucleic acid amplification (NAA)-based methods, including polymerase chain reaction (PCR) and isothermal amplification techniques, represent the forefront in diagnosing pathogens due to their rapid, sensitive, and high throughput capabilities. Although PCR and its derivatives are extensively employed for detecting various pathogens, including *H. pylori*, their utility is limited by the complexity and cost of thermocycler equipment and the need for specialized operators (Gingras and Maggiore, 2020; Liu et al., 2023; Rimbara et al., 2013; Saez et al., 2012). As a result, isothermal-based nucleic acid amplification methods, such as multiple cross displacement amplification (MCDA), loop-mediated isothermal amplification (LAMP), and recombinase polymerase amplification (RPA), were widely deployed for pathogen detection (Bogaerts et al., 2013; Saez et al., 2012). Their independence from complex equipment and compatibility with various detection formats—such as lateral flow biosensors (LFB), colorimetric readouts, and fluorescence signals—make them ideally suited for point-of-care (POC) settings and regions with limited resources (Cao et al., 2021; Li et al., 2020; Wang et al., 2018; Zhou et al., 2023).

In this study, we developed a portable detection platform for rapid and accurate detection of *H. pylori* in gastric antrum mucosa samples by coupling MCDA with nanoparticle-based LFB, termed HP-MCDA-LFB. Here, we elaborated the basic principle of the HP‐MCDA‐LFB assay, evaluated its analytical sensitivity and selectivity, and further validated the feasibility with gastric antrum mucosa samples from patients suspected of *H. pylori* infection. Our data suggested that HP‐MCDA‐LFB assay devised here could be completed within 1 h with high degree of sensitivity and specificity, highlighting that the HP‐MCDA‐LFB assay was a potential and promising tool for rapid and reliable diagnosis of *H. pylori* infection.

## 2 Materials and methods

### 2.1 Primer design

**Figure 1:**
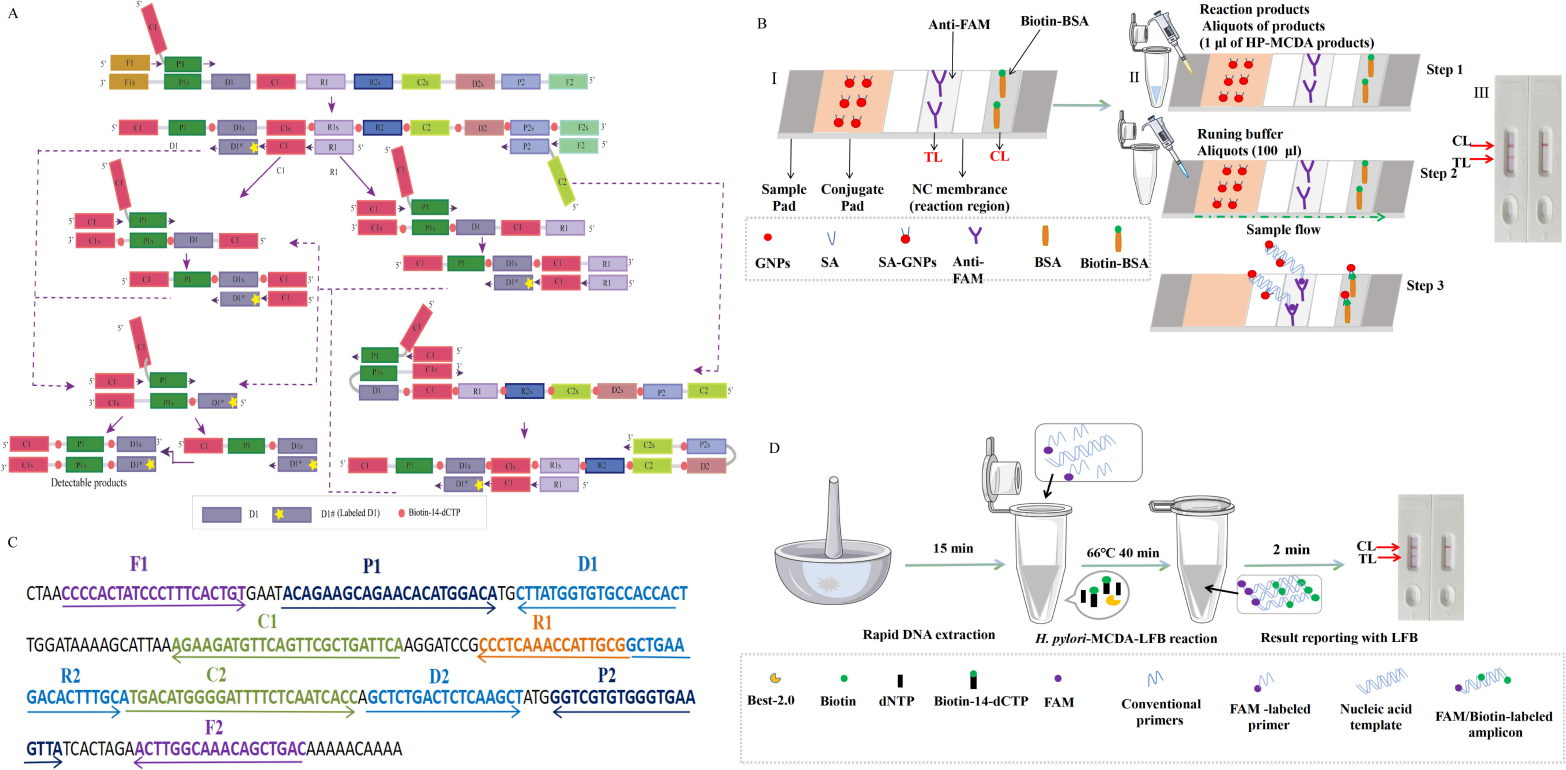
Schematic diagram of HP-MCDA‐LFB assay’s principle. **(A)** Principle of MCDA assay with D1^#^ and biotin‐14‐dCTP. D^#^ of HP‐MCDA reaction was labeled with FAM at the 5′ end and of the central-MCDA reaction with digoxin. **(B)** The detection principle of LFB for visualization of HP‐MCDA products. I, Structure diagram of LFB. II, Principle and steps of LFB for detection of HP‐MCDA products. III, Analysis of the HP‐MCDA‐LFB results: a positive result for *H. pylori* strains (TL and CL appear on the detection region); a negative result (only the CL appears on the LFB). ① a positive result for Central MPXV strains (TL1, TL2, and CL appear on the detection region); ② a positive result for Central African MPXV strains (TL2 and CL appear on the detection region); and ③ negative result (only the CL appears on the LFB). **(C)** The sequences, positions, and modifications of the gene. The right and left arrows are sense and complementary DNA sequences, respectively, which were used in this study. **(D)** Workflow of the HP‐MCDA‐LFB assay. The whole assay could be completed within 1 h, including 15 min for DNA extraction, 40 min for HP-MCDA reaction, and 5 min for results reporting. LFB, lateral flow biosensor; MCDA, multiple cross displacement amplification; HP, *H. pylori*.

The design of *H. pylori*-specific primers for MCDA reaction was performed based on the nucleotide sequence of *ureB* gene (accession no. CP003904) using the Primer Premier Version 5.0 software according to the principle of MCDA (Wang et al., 2015). The MCDA primers were composed of 10 primers targeting 10 distinct regions of target sequence, including a pair of cross primers (CP1 and CP2), a pair of displacement primers (F1 and F2), and three pairs of amplification primers (C1 and C2, D1 and D2, and R1 and R2). For biosensor detection, one amplification primer D1 was additionally labeled a fluorescein isothiocyanate (FAM) at the 5′ end (termed D1^#^). Sequences of all the primers are shown in **Table 1**. Details of primer sequences, locations, and modifications are shown in **Figure 1C**, and all the primers were synthesized and purified at HPLC purification grade by Tianyi‐Huiyuan Biotech. Co, Ltd. (Tianjin, China).

**Table 1:** Primers used in this study.

Primers ^a^	Sequence(5′-3′)	Length^b^
Hp-4-F1	CCCACTATCCCTTTCACTGT	20 nt
Hp-4-F2	GTCAGCTGTTTGCCAAGT	18 nt
Hp-4-CP1	TGAATCAGCGAACTGAACATCTTCT-ACAGAAGCAGAACACATGGA	46 mer
Hp-4-CP2	TGACATGGGGATTTTCTCAATCACC-TAACTTCACCCACACGACC	45 mer
Hp-4-C1	TGAATCAGCGAACTGAACATCTTCT	25 nt
Hp-4-D1^#^	FAM-AGTGGTGGCACACCATAAG	19 nt
Hp-4-R1	CGCAATGGTTTGAGGG	16 nt
Hp-4-C2	TGACATGGGGATTTTCTCAATCACC	25 nt
Hp-4-D2	GCTCTGACTCTCAAGCT	17 nt
Hp-4-R2	GCTGAAGACACTTTGCA	17 nt

a D1^#^, 5′-labeled with FAM when used in MCDA-LFB assay.

b mer, monomeric unit; nt, nucleotide.

### 2.2 Reagents and apparatus

The universal isothermal amplification kits, biotin‐14‐dCTP, visual detection reagent (VDR), and LFB strips were obtained from HuiDeXin Biotechnology (Tianjin, China), and the article numbers are HC-300-L, HT10005, and HT10004. The DNA extraction kits were purchased from TransGen Biotech (Beijing, China, HT0600). Real-time turbidity (LA-320C) was provided by Eiken Chemical Co., Ltd (Japan).

### 2.3 Preparation of pathogens and clinical samples

A total of 40 gastric antrum mucosa samples collected from patients admitted at the Department of Gastroenterology, Capital Institute of Pediatrics from September 2021 to March 2022 were employed in this study. A total of 17 of the 40 samples were diagnosed as *H. pylori* infection by the positive results of urea breath test or stool antigen combining with rapid urease test (RUT), while the remainder were considered as functional gastrointestinal disorder with an *H. pylori*-negative result. Clinical samples were taken from the minor curvature of the gastric antrum and one piece from the greater curvature of the gastric body (the biopsy location of the gastric antrum was within 5 cm from the pylorus opening, and the sampling location of the stomach body was within 8 cm from the cardia). Genomic DNA of the 40 clinical samples were extracted and purified using the commercially available kit (Beijing Transgen Biotech Co., Ltd.) as per the manufacturer’s instruction. The extract nucleic acid was stored at −20°C for subsequent use. In addition, 29 non-*Helicobacter* pathogens obtained from the Chinese Center for Disease Control and Prevention (CDC) were processed as the clinical samples, and the extracted genomic DNA samples were employed for the specificity analysis of the HP-MCDA-LFB (**Table 2**).

**Table 2:** Bacterial strains used in the current study.

No.	Bacteria	Source^b^	No. of strains	HP-MCDA-LFB result^c^
1	*Helicobacter pylori*	ICDC	1	P
2	*Helicobacter pylori*	ICDC	1	P
3	*Bacillus cereus*	ICDC	1	N
4	*Citrobacter* spp.	ICDC	1	N
5	*Candida albicans*	ICDC	1	N
6	*Corynebacterium striatum*	ICDC	1	N
7	*Candida albicans*	ICDC	1	N
8	Enteroaggregative *E. coli* ^a^	ICDC	1	N
9	Enteroinvasive *E. coli*	ICDC	1	N
10	Enteropathogenic *E. coli*	ICDC	1	N
11	Enterotoxigenic *E. coli*	ICDC	1	N
12	*Enterobacter cloacae*	ICDC	1	N
13	*Enterococcus faecium*	ICDC	1	N
14	*Klebsiella pneumoniae*	ICDC	1	N
15	*Listeria monocytogenes*	ICDC	1	N
16	*Mycobacterium tuberculosis*	ICDC	1	N
17	*Pseudomonas aeruginosa*	ICDC	1	N
18	Pyogenic streptococcus	ICDC	1	N
19	*Plesiomonas shigelloides*	ICDC	1	N
20	*Pertussis bacillus*	ICDC	1	N
21	*Rothia* spp.	ICDC	1	N
22	*Staphylococcus haemolyticus*	ICDC	1	N
23	*Staphylococcus epidermidis*	ICDC	1	N
24	*Streptococcus meningococcus*	ICDC	1	N
25	*Staphylococcus aureus*	ICDC	1	N
26	*Streptococcus pneumonia*	ICDC	1	N
27	*Saprophytic bacteria*	ICDC	1	N
28	*Salmonella* spp.	ICDC	1	N
29	*Streptococcus salivarius*	ICDC	1	N
30	*Shigella boydii*	ICDC	1	N
31	*Vibrio alginolyticus*	ICDC	1	N

a E. coli, Escherichia coli.

b ICDC, National Institute for Communicable Disease Control Disease Control and Prevention, Chinese Center for Disease Control and Prevention.

c P, positive; N, negative.

### 2.4 The standard HP-MCDA reaction assay

As per the previous studies (Wang et al., 2015, 2018), the HP-MCDA reaction was conducted in a 25-µl amplification mixture, containing 0.4 µM each of displacement primer (F1 and F2), 0.8 µM each of amplification primer (C1, C2, R1, R2, D1^#^, and D2), 1.2 µM each of cross primer (CP1and CP2), 12.5 µl 2× reaction mixture, 1.5 µl VDR, 1 µl *Bst* 2.0 DNA polymerase, 1 µl DNA template from pure culture or 5 µl from clinical specimens, and moderate distilled water (DW) to a final volume of 25 µl. The MCDA reaction mixtures were incubated at 66°C for 40 min followed by 105°C for 5 min to stop the amplification. Genomic DNA of *H. pylori* was used as positive control, that of *Listeria innocua* (a resident of food, which may transiently exist in the stomach) as negative controls, and DW as blank control. The MCDA reaction results were determined according to the dynamic curve of real-time turbidity and the color change of the final reaction tube. Typically, turbidity value higher than 0.1 or a light blue color of reaction mixture indicated a positive result, while that lower than 0.1 or colorless implied a negative one.

### 2.5 Biosensor detection of HP-MCDA products

For biosensor detection of the products of HP-MCDA reaction, LFB strip was utilized. In detail, an aliquot of 1 μl MCDA product was added to the sample pad region of LFB (**Figure 1B**, II, step 2), followed by addition of an aliquot of 100 μl running buffer (10 mM PBS, pH 7.4 with 1% Tween 20) to the same region (**Figure 1B**, II, step 2). After 5 min, the result was indicated by the presence or absence of red band in the NC membrane region (**Figure 1B**, III), with red bands occurring in both the testing line (TL) and control line (CL) as positive result and only a red band in CL as negative.

### 2.6 Optimization of the HP-MCDA-LFB assay

The performance of the HP-MCDA-LFB assay may be influenced by the MCDA reaction temperature, reaction time, and the volume of MCDA products for LFB detection. The optimal reaction temperature of HP-MCDA reaction was determined by performing the reactions at temperatures of 60°C–67°C at 1°C intervals for 40 min. The optimal reaction time was examined by implementing MCDA reactions for 10–40 min (with 10 min interval), respectively. The optimal volume for LFB test was evaluated by adding varied volumes (0.25 µl, 0.5 µl, 1 µl, 1.5 µl, and 2.0 µl) of HP-MCDA products to the sample region of LFB strip and reporting the result within the same period of time.

### 2.7 Sensitivity and specificity evaluation of the HP‐MCDA‐LFB assay

Tenfold serial dilution of the genomic DNA of *H. pylori* (600 pg μl^−1^ to 60 fg μl^−1^) was prepared to determine the analytical sensitivity and limit of detection (LoD) of the MCDA-LFB assay for *H. pylori* detection. In parallel, MCDA products of the serial dilutions of genomic DNA of *H. pylori* was visually reported with VDR. Each test was implemented with three replicates.

### 2.8 Specificity evaluation of the HP-MCDA-LFB assay

The specificity of the HP-MCDA-LFB assay was evaluated using genomic DNA templates from 29 bacterial pathogens. After amplified under the conditions mentioned above, the obtained products were interpreted by both VDR and LFB detection formats. Each sample was tested independently at least three times.

### 2.9 Clinical feasibility verification of the HP-MCDA-LFB assay

In order to further validate the feasibility of the HP-MCDA-LFB technology in clinical *H. pylori* detection, gastric antrum mucosa samples from patients suspected of *H. pylori* infection at Children’s Hospital Capital Institute of Pediatrics were collected. These samples were detected by RUT method previously, a common rapid diagnostic method of *H. pylori* via detecting the activity of bacterial enzyme urease. Detail information of all the samples was listed in **Table 3**. Genomic DNA extracted from the rest of these samples was deployed to HP-MCDA-LFB test. Results obtained through both methods were compared to confirm the clinical application potential of the HP-MCDA-LFB assay.

**Table 3:** Baseline characteristics without *H. pylori* infection and with *H. pylori infection*.

	without Hp infection (n=23)	Hp infection (n=17)	*p-*value
Age [mean (SD)]	9.65 (2.78)	11.39 (2.87)	0.061
Medical history [mean (SD)]	14.17 (20.07)	15.71 (25.53)	0.833
Girl (n,%)	13 (56.5)	6 (35.3)	0.313
Boys (n,%)	10 (43.5)	11 (64.7)	
Stomachache (n,%)	14 (60.9)	15 (88.2)	0.079
Nausea (n,%)	9 (39.1)	2 (11.8)	0.079
Acid reflux (n,%)	4 (17.4)	0 (0.0)	0.123

Age, year. Medical history, mon.

## 3 Result

### 3.1 Schematic illustration of the HP-MCDA-LFB assay

The principle of the MCDA-LFB method for *H. pylori* detection is shown in **Figure 1A**. Briefly, the MCDA reaction was carried out as previously described, which used only a DNA polymerase with strand-replacement activity (Bst 2.0) and a set of 10 primers specifically spanning 10 different regions of the target sequence to achieve exponential amplification of the analytes. The MCDA assay was initiated by the cross primer CP1 annealing the target sequence, then replaced by the newly synthesized strand primed with F1, leading to release of the strand primed with CP1, which was employed as templates of subsequent amplification. Within a short time, plenty of strands complementary to the primer D1^#^ were produced, resulting in substantial double strands primed with D1^#^ generated. Particularly, biotin-labeled cytosine deoxynucleotide (biotin-14-dCTP) was additional added in the reaction mixture, leading to biotin labeled in the amplification products. As a result, the D1^#^ primed products contained both FAM and biotin labels and were considered as the detectable targets, which could be captured and visually inspected with LFB strips.

### 3.2 Principle of biosensor detection of HP-MCDA products by LFB

Details of biosensor detection of HP-MCDA products with LFB are shown in **Figure 1B**. The LFB strip was adapted for use by using anti-fluorophore antibodies (anti-FAM) to capture FAM-containing amplicons and using streptavidin-coated dyed (crimson red) polymer nanoparticles (SA-GNPs) to bind biotin-containing amplicons for readout visualization. As previously described (Wang et al., 2018; Zhou et al., 2023), SA-GNPs were deposited on the conjugate pad of the LFB strip; anti-FAM and biotinylated bovine serum albumin (biotin-BSA) were on the TL and CL regions of NC membrane, respectively. If the target analyte was present, the double-labeled (FAM and biotin-containing) products will flow down the strip and eventually captured by anti-FAM and visualized via SA-GNPs with red bands occurring in both TL and CL regions, while the absence of target amplicons only appeared as red band only in CL region due to the interaction between biotin-BSA and SA-GNPs.

### 3.3 Effectiveness confirmation of the primers for HP-MCDA-LFB assay

**Figure 2:**
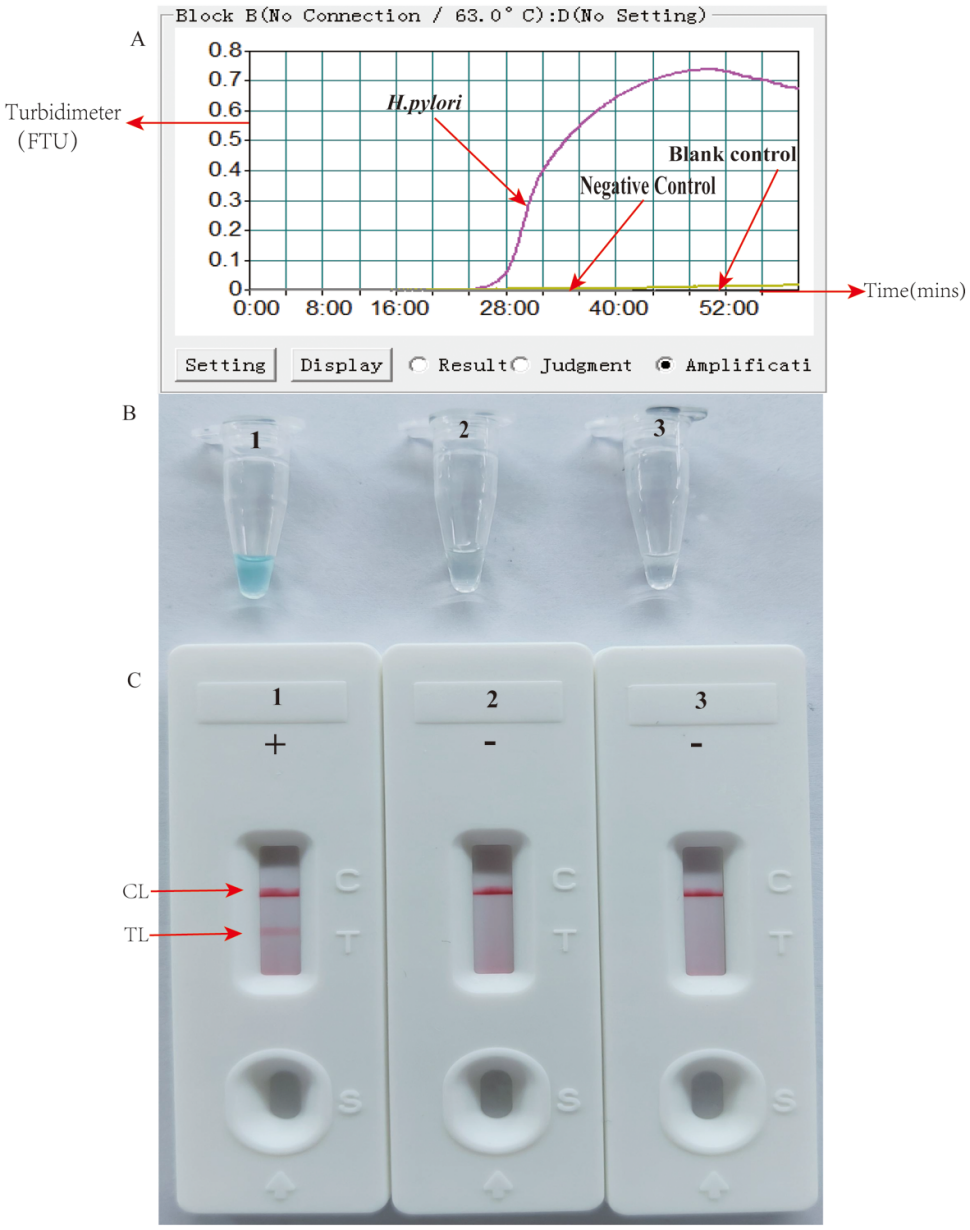
Detection and confirmation of HP-MCDA-LFB assay for *H. pylori* detection. Amplification products were analyzed by measuring turbidity in real time **(A)**. The ordinate represents the turbidimeter (FTU), the abscissa axis is reaction time (min). VDR **(B)** and LFB **(C)**. Tube 1/biosensor 1, positive amplification of *H. pylori*; tube 2/biosensor 2, negative control of *Listeria innocua*; tube 3/biosensor 3, blank control of double distilled water (DW).

Primer set feasibility for the HP-MCDA-LFB assay was determined by performing *H. pylori* MCDA reaction and reporting the results with LFB strip along with real-time turbidimeter and VDR. As shown in **Figure 2A**, by real-time turbidimeter, it was observed that only the positive control reaction reached a turbidity value higher than 0.1 within a short time, while the negative and blank controls all displayed blunted curves within the whole incubation time; with VDR, the tube of positive control mixture showed light green in color while that of negative and blank controls were colorless (**Figure 2B**); on the LFB strips, positive control reaction products induced two red bands in the TL and CL region, simultaneously, while the others only exhibited a red band in the CL region (**Figure 2C**). Data from the three detection formats demonstrated the effectiveness of the selected primer and its feasibility in the HP-MCDA-LFB assay for *H. pylori* detection.

### 3.4 Optimal reaction conditions of HP-MCDA-LFB assay

**Figure 3:**
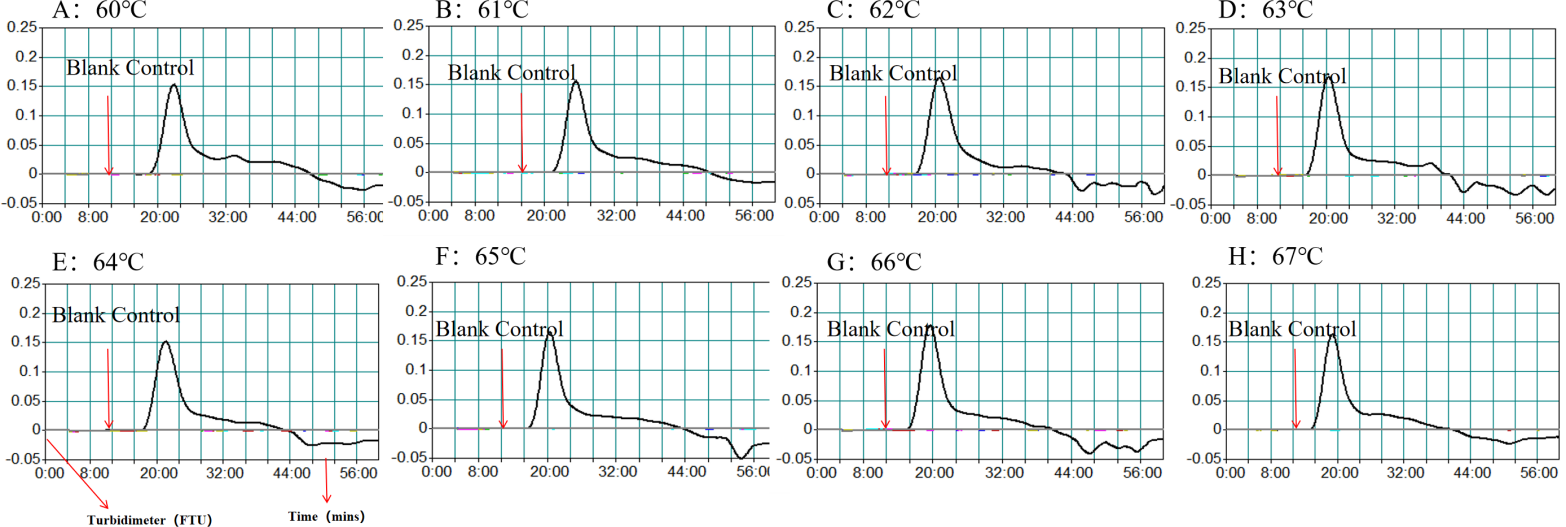
Optimization of amplification temperature for MCDA assay. The standard MCDA reactions for detecting *H. pylori* was monitored by measuring turbidity in real-time manner. The threshold value was 0.1, and a turbidity value > 0.1 was considered a positive result. Eight kinetic graphs **(A–H)** were obtained at various temperatures (60°C–67°C, 1°C intervals) with target pathogens DNA at the level of 60 fg µl^−1^.

**Figure 4:**
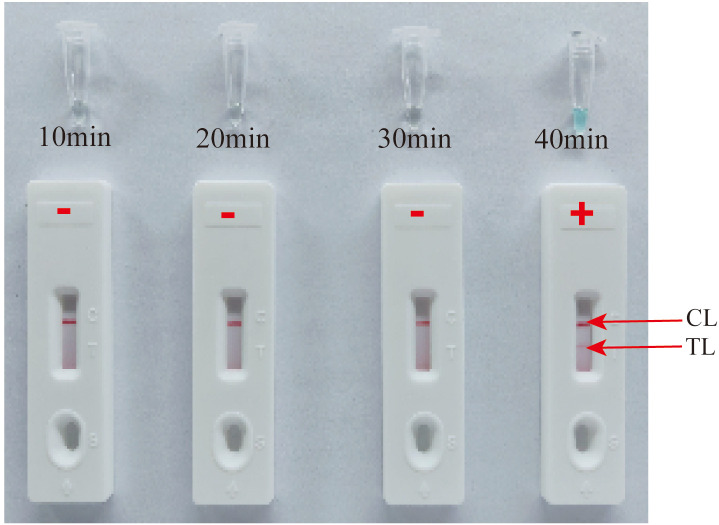
Optimization of reaction time for HP-MCDA-LFB assay. Four different reaction times (10 min, 20 min, 30 min, and 40 min) were tested and compared using *H. pylori* pathogens at a concentration of 60 fg at the optimal amplification temperature (66°C).

**Figure 5:**
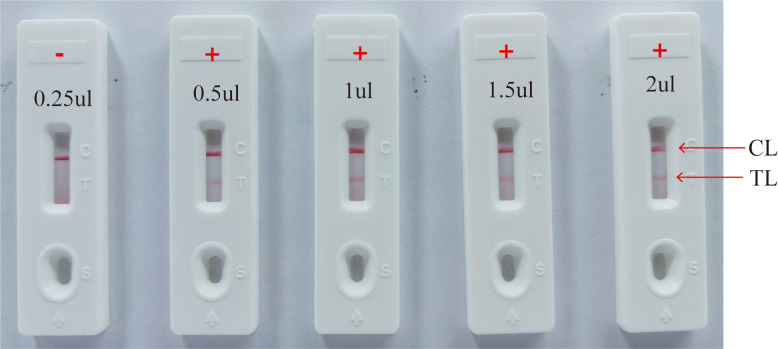
Optimization of amplicon content for LFB detection. Different volumes (0.25 µl, 0.5 µl, 1 µl, 1.5 µl, and 2.0 µl) of HP-MCDA amplicon were extracted and titrated into LFBs to verify the sufficient content for effective detection by LFB assay; “+” positive; “−” negative.

Efficiency of the HP-MCDA assay at temperatures 60°C–67°C were exhibited in **Figure 3**. According to the kinetic curves, it was observed that reactions at 66°C allowed for the most abundant products and the fastest speed to reach a turbidity of 0.1. Thus, 66°C was considered as the optimal reaction temperature of HP-MCDA reaction. Additionally, after amplifying LoD level of genomic DNA of *H. pylori* (60 fg) at 66°C for 10 min, 20 min, 30 min, and 40 min, respectively, the products were detected in formats of VDR and LFB. As shown in **Figure 4**, the shortest time required to detect LoD levels of genomic DNA of *H. pylori* was 40 min. As a result, the whole detection procedure for HP diagnosis, including rapid template extraction (15 min), MCDA reaction (40 min), and result reporting using LFB (5 min), could be finished within 1 h (**Figure 1D**). Then, the optimal volume of products for LFB detection was determined by loading 0.25, 0.5, 1, 1.5, and 2 µl of amplification products to the sample pad region of LFB strip, respectively. As displayed in **Figure 5**, the LFB strips loading 0.5–2 µl of product all showed two red bands at CL and TL regions within 5 min, while the one with 0.25 μl products showed only one red band at CL. Considering the potential of aerosol pollution, 0.5 μl of HP-MCDA product was accepted for LBF detection. In summary, the optimal reaction conditions, including a reaction temperature of 66°C, a reaction time of 40 min, and a product loading volume of 0.5 μl, were employed for the subsequent analysis.

### 3.5 Sensitivity and specificity of the HP-MCDA-LFB assay

**Figure 6:**
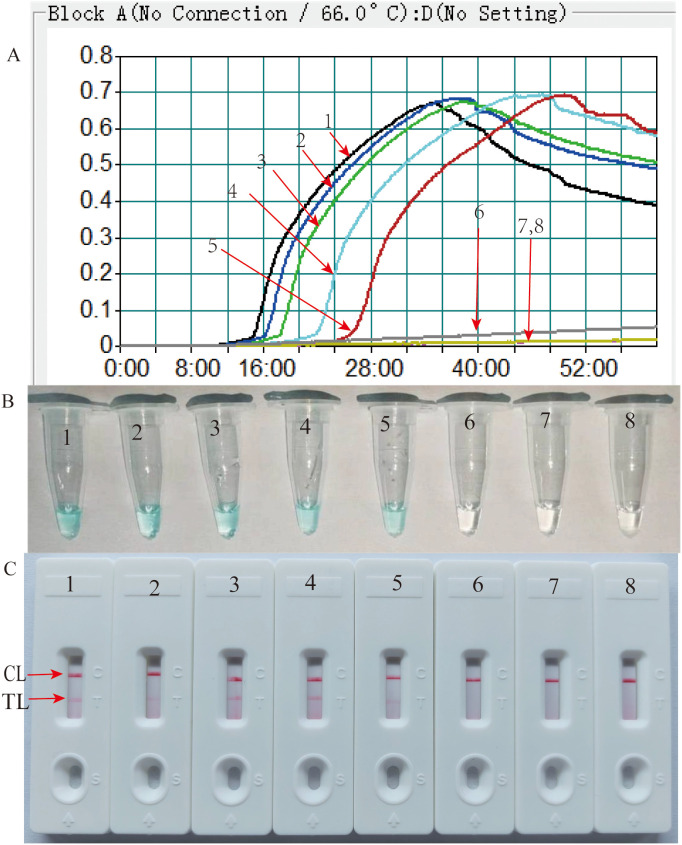
Analytical sensitivity of HP-MCDA-LFB assay. Serial dilutions (600 pg µl^−1^, 60 pg µl^−1^, 6 pg µl^−1^, 600 fg µl^−1^, 60 fg µl^−1^, and 6 fg µl^−1^) of target templates were subjected to conventional MCDA reactions, and the results were monitored by real-time turbidity **(A)**, VDR **(B)**, and LFB **(C)** formats. Biosensors A1–A7 (tubes 1–7 and signals C1–C7). A1–A7 shows different concentrations of *H. pylori* genomic templates; blank line is 600 pg µl^−1^, blue line is 60 pg µl^−1^, green line is 6 pg µl^−1^, wathet blue line is 600 fg µl^−1^, brown line is 60 fg µl^−1^, and yellow line is 6 fg µl^−1^), respectively; biosensors A8 (Tube B 8 and Signal C 8) indicated negative control (DW).

**Figure 7:**
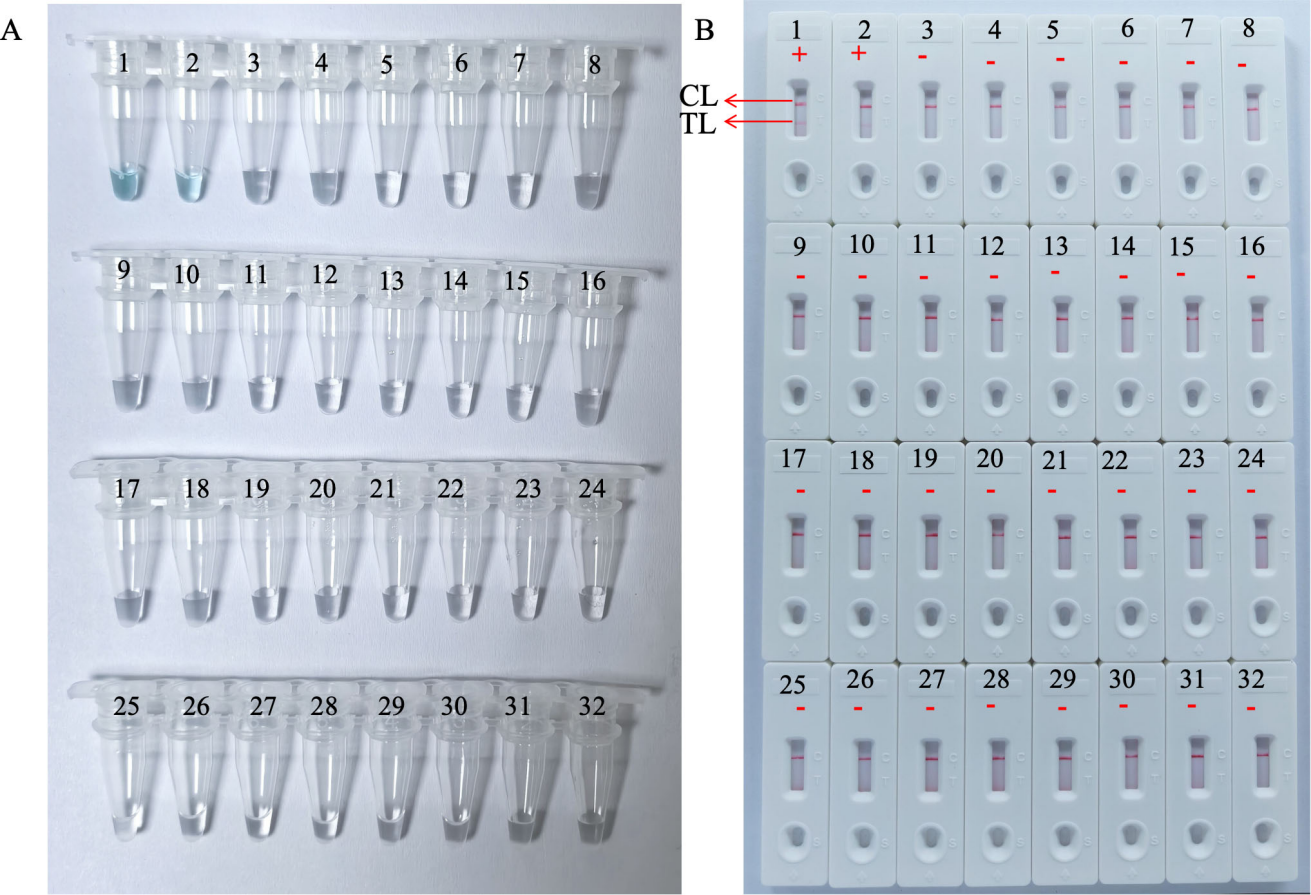
Analytical specificity of the HP-MCDA-LFB assay. The specificity of the HP-MCDA-LFB assay was evaluated using 29 non-*Helicobacter* pathogens. The amplification products was interpreted by VDR method **(A)** and LFB platform **(B)**. Tubes/LFBs 1–2 represented *H. pylori* pathogens, and 3–31 showed 29 non‐*H. pylori* pathogens, and the details are shown in **Table 3**. CL, control line; LFB, lateral flow biosensor; MCDA, multiple cross displacement amplification; HP, *H. pylori*; TL, test line.

Analytical sensitivity of the HP-MCDA-LFB was determined using the serial dilutions of genomic DNA templates of *H. pylori*. After testing each titer with three replicates, it was revealed that the detection limit of HP-MCDA-LFB assay was 60 fg μl^−1^, which was demonstrated by LFB strip exhibiting two red bands in the TL and CL regions when loaded with products from templates not less than 60 fg (**Figure 6C**). Similarly, only the reaction with at least 60 fg templates displayed a sharp curve by real-time turbidimeter (**Figure 6A**) and showed light green in color (**Figure 6B**), revealing an identical LoD level to HP-MCDA-LFB assay. For the purpose of specificity analysis, 29 non-*Helicobacter* pathogens were detected. As envisaged, all the LFB strips loaded with MCDA products from non-*Helicobacter* pathogens showed red band only in the CL regions, while the positive control displayed two red bands in the TL and CL regions of LFB strip (**Figure 7**), indicating a 100% specificity of HP-MCDA-LFB assay in *H. pylori* detection.

### 3.6 Feasibility assessment of the HP-MCDA-LFB assay in clinical samples

**Figure 8:**
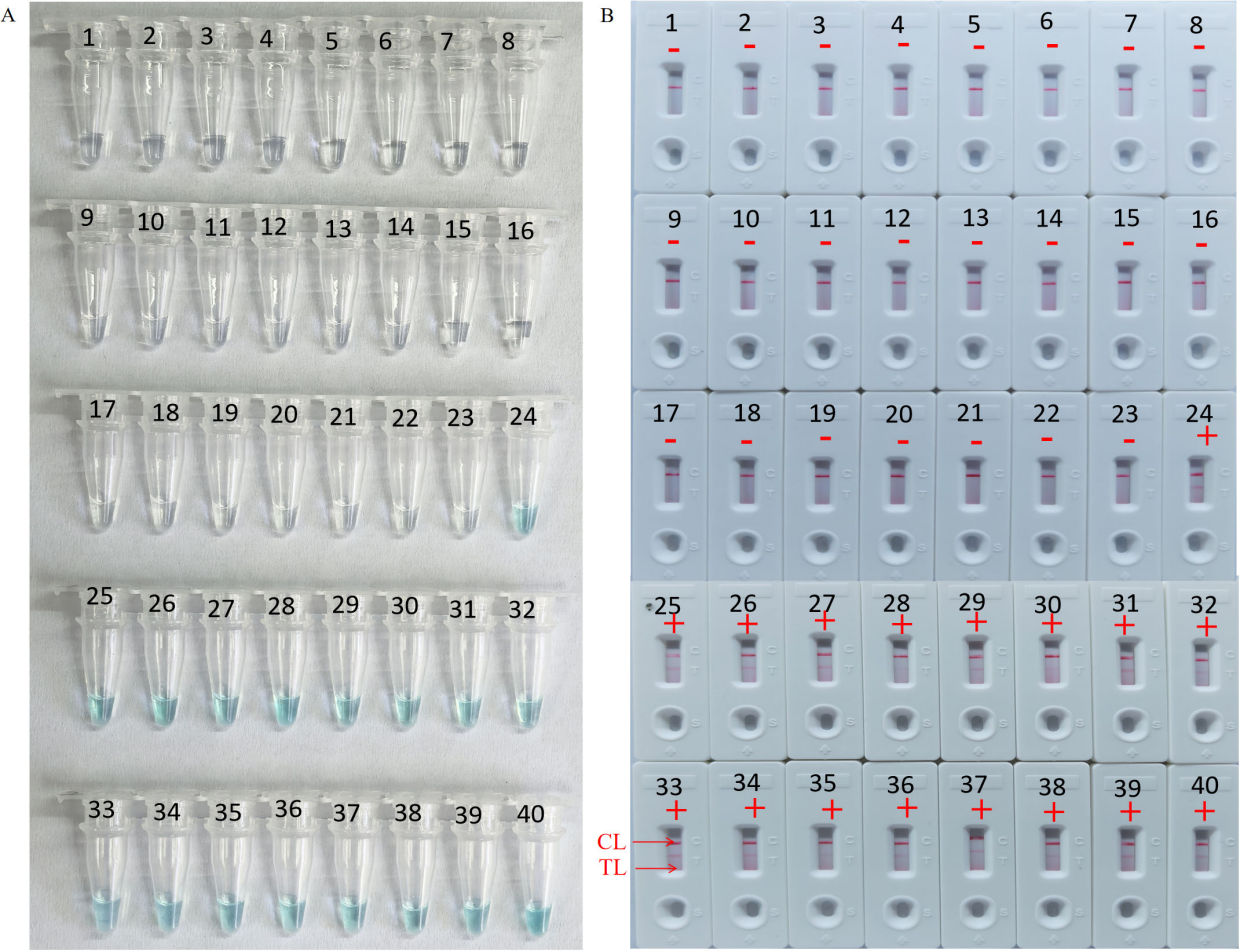
Clinical feasibility assessment of the HP-MCDA-LFB assay. The amplification products was interpreted by VDR method **(A)** and LFB platform **(B)**. Tubes 1–23 using VDR method represented clinical samples that were not considered *H. pylori* infection by rapid urease tests, Tubes 24–40 using VDR method represented clinical samples that were considered *H. pylori* infection by rapid urease tests. B1–23 using LFBs method represented clinical samples that were not considered *H. pylori* infection, B24–40 using LFBs method represented clinical samples that were considered *H. pylori* infection. CL, positive control; TL, negative control; “+” positive; “−” negative.

To further validate the feasibility of HP-MCDA-LFB as a diagnostic tool for *H. pylori* in clinical settings, we examined 20 endoscopically suspected of *H. pylori* infected gastric sinus tissues and 20 gastric sinuses with normal endoscopic mucosa using both HP-MCDA-LFB and RUT. As a result, 17 *H. pylori*-positive samples diagnosed by RUT were tested positive for *H. pylori* by HP-MCDA-LFB, and the 23 URT *H. pylori*-negative samples were negative in the HP-MCDA-LFB assay as well (**Figure 8**), demonstrating high degree of agreement between the two methods. The results implied that the HP-MCDA-LFB method was an accurate and sensitive screening tool for *H. pylori* infection and indicated great potential to be widely used in clinical laboratories and basic research.

## 4 Discussion

*H. pylori* represents one of the most widespread bacterial infections all over the world, affecting half of the world’s population with high morbidity and mortality (Malfertheiner et al., 2017). Although the overall prevalence of *H. pylori* infection has declined in recent years with living standards improved, reported morbidity and mortality associated with gastric cancer in adults remain high (Hooi et al., 2017). In addition, *H. pylori* has been reported increasingly resistant to clarithromycin and metronidazole, which were essential for the eradication of *H. pylori* (Zheng et al., 2022). The increasing antibiotic resistance rate has given rise to treatment failure, further aggravating the global burden of *H. pylori*-related gastric complications. Accurate and early diagnosis of *H. pylori* infection paved a critical way toward successful eradication of *H. pylori* and alleviating the progress of antimicrobial resistance.

Because of population growth and because of reinfection and recrudescence due to unsuccessful eradication, *H. pylori* infection rates persisted or even increased over the past three decades in the world (Hu et al., 2017). The clinically commonly used techniques for the detection of *H. pylori* to diagnose *H. pylori*-induced complications and evaluate the outcomes of *H. pylori* eradication included urea breath test (UBT), RUT, and bacterial culture. UBT is the most widely recommended method for *H. pylori* detection and for eradication effort assessment after antibiotic therapy (Chen et al., 2023). Due to the noninvasive property, however, the result was vulnerable to the drug used and endogenous carbon dioxide production. Therefore, a simple, easy-to-operate, rapid, and sensitive test device remains highly desirable for primary care providers to detect *H. pylori* infection in resource-limited areas.

The newly devised HP-MCDA-LFB assay, an isothermal nucleic acid detection platform targeting the critical virulence factor coding gene *ureB* (Liu et al., 2023, 2023; Tanahashi et al., 2000), exhibited excellent performance in terms of sensitivity and specificity and was promising in the rapid, simple, and accurate diagnosis of *H. pylori* infection. Our findings suggest that the newly designed Hp-MCDA-LFB assay has high sensitivity and specificity and is well suited for implementation in primary care settings.

The newly developed HP-MCDA-LFB assay is a user-friendly, simple, and portable diagnostic tool for *H. pylori* detection. The HP-MCDA-LFB assay utilized MCDA technique to amplify target sequences and LFB for results indication. MCDA has been extensively deployed to detect nucleic acid of various pathogens due to the advantages of instrument-free, high amplification efficiency, and ultra-accuracy. Typically, the MCDA assay could achieve exponential increase in target analytes at a constant temperature (60°C–67°C) for 30–40 min using simple equipment such as metal baths, water baths, or hot water cups. In addition, the nanoparticle-based LFB allows for intuitive and objective interpretation of amplification results without expensive and complex laboratory conditions. Of note, the LFB detection of target nucleic acid in this study required only one labeled primer along with biotin-14-dCTP rather than two labeled primers, which lowered the design difficulty and cost (Chen et al., 2022; Jiang et al., 2021; Li et al., 2020; Wang et al., 2016, 2015; Zhou et al., 2023). The entire detection process, including rapid sample processing (15 min), MCDA reaction (40 min), and LFB detection (within 5 min), can be completed within 1 h. The fluorescence detection method in this study does not rely on any PCR system, but on a simple blue light device. In addition, the MCDA reaction does not include multiple denaturation and annealing cycles, which avoids the need for complex and expensive PCR equipment. Meanwhile, the cost of one MCDA-LBF reaction was less than $10 (Wang et al., 2017, 2015, 2016). Hence, the HP-MCDA-LFB assay is a simple, rapid, user-friendly, and cost-effective method that had great potential in resource-limited areas for the diagnosis of *H. pylori* infections.

The HP-MCDA-LFB assay demonstrated excellent performance in the detection of *H. pylori*. Prior to detection ability analysis, related parameters, including reaction temperature and time, and detection volume, were determined in order to achieve enhanced performance and less aerosol pollution. As a result, the optimal reaction conditions were examined as reacting at 66°C for 40 min with a volume of 0.5 µl products for LFB detection. Under the optimal conditions, the HP-MCDA-LFB assay was able to detect low to 60 fg (~56 copies) genomic DNA of *H. pylori*, which was more sensitive than the loop-mediated isothermal amplification-lateral flow dipstick (LAMP-LFD) method and polymerase chain reaction (PCR) (Liu et al., 2023), but less sensitive than the CRISPR-based detection platform (Dai et al., 2022). It was supposed that the detection method itself and/or the detection targets resulted in difference in detection sensitivity. Thus, further comparison and development need to be implemented for better diagnostic efficiency. The result of specificity analysis of the HP-MCDA-LFB method displayed no cross-reaction of this method with non-*Helicobacter* pathogens, indicating the reliability of HP-MCDA-LFB for *H. pylori* detection. In addition, the MCDA-LFB assay excels in detecting clinical *H. pylori* infection. In our investigation, the assay accurately identified *H. pylori* infection in 17 tissue mucosa-extracted DNA samples identified as having *H. pylori* infection. Meanwhile, it correctly excluded the presence of *H. pylori* in 23 tissue mucosa samples not caused by *H. pylori* infection. Consistent results with those from the combined RUT diagnostic approach emphasize the reliability of the HP-MCDA-LFB assay in clinical *H. pylori* detection.

Overall, the HP-MCDA-LFB demonstrates remarkable sensitivity and specificity, enabling visual detection of *H. pylori* infection with basic equipment. It is capable of detecting as little as 60 fg (~56 copies) genomic DNA of *H. pylori* with 100% specificity for clinical samples. Given its simplicity, sensitivity, and specificity, the HP-MCDA-FRT assay has great potential as a powerful tool for *H. pylori* detection in the primary care setting.

Several drawbacks yet to be addressed included the following: (1) samples for clinical validation in this study just involved the gastric sinus tissue, thus more types of samples associated with *H. pylori* infection are yet to be detected such as stool and saliva, which could be accessed noninvasively and more favored by the patients; (2) the LFB detection step in this study required opening the reaction tube, which increases the risk of carrying contamination and the possibility of false-positive result, thus methods integrating amplification and detection into one step are yet to be developed; and (3) the viable but non-culturable (VBNC) state of *H. pylori* in water reservoirs remains a high risk factor of public health, thus confirmation of the effectiveness of the new method in water reservoirs will be highly appreciated.

## 5 Conclusion

In summary, we combined isothermal MCDA with nanoparticle-based LFB to establish an HP-MCDA-LFB diagnostic method for *H. pylori* detection. The analyzed data showed that the HP-MCDA-LFB method is a user-friendly, rapid, sensitive, and specific method for diagnosing *H. pylori* infections and can be applied in the clinical setting. The HP-MCDA-LFB assay can be performed within 1 h without the need for complex instruments and skilled technicians. Therefore, the HP-MCDA-LFB test developed in this study is an effective tool for the rapid and reliable diagnosis of *H. pylori* infection in both centralized laboratory and rural areas.

## Data Availability

The original contributions presented in the study are included in the article/supplementary material. Further inquiries can be directed to the corresponding authors.
